# Assessment of Knowledge about Healthcare Risk Waste Management at a Tertiary Hospital in the Northern Cape Province, South Africa

**DOI:** 10.3390/ijerph18020449

**Published:** 2021-01-08

**Authors:** Mokete Motlatla, Thelmah Xavela Maluleke

**Affiliations:** Department of Public Health, Faculty of Health Sciences, University of Fort Hare, East London 5201, South Africa

**Keywords:** healthcare risk waste, health professionals, knowledge

## Abstract

This study aimed at assessing the knowledge about healthcare risk waste (HCRW) management among doctors, professional nurses, pharmacists and laboratory technicians, in accordance with National Environmental Management, Waste Act 59 of 2008, Constitution of South Africa and sustainable development goals (SDG). The quantitative cross-sectional study was conducted, using self-administered questionnaires and stratified random sampling was used. Data was analyzed using the descriptive and inferential statistics. One hundred and forty-four participants were included in the study. The majority 90.28% of the participants were aged 19–50 years, females (71%), professional nurses (36%), and they had 1–10 years of experience (71%). The health professionals were knowledgeable of sharps waste (89%), slightly over (52%) knew anatomical waste, whereas (27%) and (17%) knew radioactive and cytotoxic waste, respectively. Health professionals (92%) agreed that the sharps-waste container should be disposed of in a yellow bin container, at least (63%) and (27%) agreed that red liner and box should be used for both infectious non anatomical waste and for cytotoxic waste. The null hypothesis was tested on knowledge versus age, profession and gender, and evidence against it was found on waste storage period in all three variables where chi-square and Fisher exact *p*-values were less than the 5% significant level. More attention should be directed towards similar HCRW management training at the hospital for all health professionals and behavior modification. The hospital management must ensure that HCRW-trained health professionals and HCRW management officials put into practice what they have learnt.

## 1. Introduction

Health care risk waste (HCRW) is the hazardous portion of healthcare waste that includes wastes from maternity care, soiled dressings, diagnosis, blood and any diagnostic samples, infectious waste, sharps, syringes, pharmaceutical waste, body parts and radioactive materials. It is a major contributor to environmental pollution if it is incorrectly managed by healthcare workers, waste management, disposal services, and handlers at healthcare facilities. The indiscriminate and erratic handling, management and disposal of HCRW within the healthcare facilities have been widely acknowledged as sources of infections within the hospitals and communities [1]. These improper handlings of HCRW are perceived by both the public and healthcare workers as poor standards of healthcare and waste management in hospitals [2]. Although healthcare facilities worldwide generate high volumes of waste, only 15% of this waste is hazardous and considered to be HCRW [2]. The generation of high volumes of HCRW in healthcare facilities is due to improper segregation of HCRW, which contaminates non-hazardous healthcare waste (HCW) and converts all into HCRW [3]. These high volumes of HCRW generation increases the chances of infections such as HIV, Hepatitis B and C and exposure to hazardous medical waste among hospital staff, patients, informal recyclers (scavengers) and employees at the landfill site [4,5,6]. It can also increase costs of running the hospitals due to litigations and cleaning costs related to exposure to hazardous waste [7]. Correct waste management principles and segregation, and compliance with waste management policies, guidelines and standards reduce the amount of HCRW generation in healthcare facilities and; therefore, prevent the generation of high volumes of HCRW, and exposures to hazardous waste in hospitals and communities [2]. 

A number of studies have shown some inadequacies and non-compliance with waste management principles at hospitals and landfill sites in the developing countries including South Africa [8]. In South Africa the management of HCRW is regulated by the National Health Act no 61 of 2003, National Environmental Management Act no. 107 of 1998, National Environmental Management Waste Act no. 59 of 2008, National Core Standards, the Constitution of South Africa and Norms and Standards Regulations published in terms of the National Health Act under GN 67 in Government Gazette No. 41419 of 2 February 2018, which require that all healthcare facilities must maintain safe environments that minimize the risks of disease outbreaks and transmission of infections to patients to healthcare staff and visitors [9,10,11,12,13]. These national legislations require that waste generation should be avoided and if it cannot be avoided, it should be minimized, recycled or reused or disposed of in accordance with the municipality, national and international policies and regulations and in a manner that is environmentally-friendly [9,10,11,12,13]. However, studies in South Africa, including the National Core Standards, Office of the Standards Health Compliance and Auditor General reports, indicate major challenges in the management of HCRW in the different levels of healthcare facilities in the different provinces [3,4,5,8,14,15]. 

**Figure 1:**
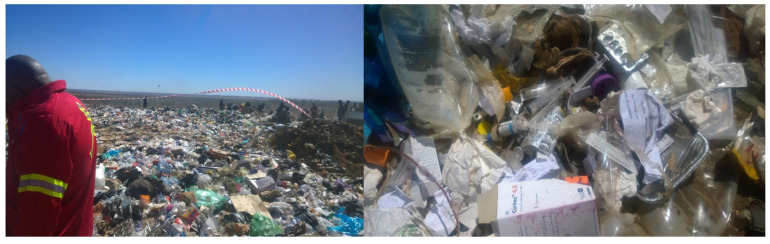
Health care waste from a tertiary hospital disposed at a local landfill site.

In 2016 evidence of non-compliance with waste management policies and principles became evident in the tertiary hospital in the Northern Cape Province, as presented in Figure 1. This was discovered during an investigation into HCRW performance standards at the hospital and local landfill sites by the Department of Health after numerous complaints from the local municipality and communities [14,15,16]. The tertiary hospital in the Northern Cape Province is a complex healthcare institution that offers highly-specialized healthcare services [14,16]. This hospital, like many other tertiary hospitals in South Africa, generate high quantities of HCRW that require diligent management and compliance with policies, guidelines, national core standards and municipal regulations for the safety of staff, patients and local communities [14,15,16]. 

Several measures have been implemented to address the crisis of indiscriminate and erratic handling and management of HCRW at the tertiary hospital in the Northern Cape Province. This resulted in a reduction of complaints during the 2017 calendar year. However, crisis around the high volumes of HCRW still remains a challenge due to the mixing of general waste with HCRW at the tertiary hospital and rendering all HCW hazardous that is still continuing [16]. This could suggest that healthcare professionals as generators and handlers of HCRW do not categorize, estimate accurately the quantities of all the categories and handle and dispose of the different categories of HCRW as required by the local, national and international policies, regulations and standards (see Figure 1, which demonstrated HCRW from a tertiary hospital dumped at a landfill site in the Northern Cape) [14]. Furthermore, these inadequacies in HCRW management raise questions on whether health professionals, as waste generators and handlers and waste disposal contractors, have the necessary knowledge and skills to implement appropriate HCRW segregation, storage and disposal practices. The lack of knowledge could be contributing to the high volumes of HCRW that are generated and the inadequate HCRW management by the tertiary hospital. It was; therefore, important to assess the knowledge of health professionals as generators and handlers of HCRW from the time it is produced, through to handling and disposal [17]. The tertiary hospital conduct training on HCRW. However, these programs are intermittent and do not form part of the induction program of the hospital to ensure all new staff members receive training. There is; therefore, a high possibility that some health professionals have not received any training on HCRW. They could; therefore, be experiencing challenges in the management of HCRW during the performance of their work activities resulting from lack of knowledge. 

The study addressed an important section of the sustainable development goals (SDGs)—goal eleven—which emphasizes the reduction of the adverse per capita environmental impact of cities, air quality and waste management by 2030, in order to improve environmental and air quality standards [18,19,20,21]. Appropriate waste management knowledge at tertiary hospitals by HCRW generators and handlers could contribute towards the achievement of this goal. The recent scourge of Coronavirus disease (COVID-19) pandemic has elevated the need for appropriate HCRW management system in this healthcare facility and the need for knowledgeable and skilled health professionals in HCRW management.

This article discusses the finding of a study that was conducted at a tertiary hospital in the Northern Cape Province of South Africa in 2019. The purpose of the study was to assess knowledge of health professionals regarding the management of HCRW, in accordance with the legislations, policies, guidelines and norms and standards at a tertiary hospital in the Northern Cape Province.

## 2. Material and Methods

### 2.1. Study Design and Study Setting

This was a cross-sectional quantitative descriptive study conducted among healthcare professionals in different wards, units and workstations at the tertiary hospital of the Northern Cape Province. The tertiary hospital is a 534 bedded hospital with a 30 bedded rehabilitation center. It has 21 heads of specialty programs, with at least 745 permanent staff of different categories and 202 permanent health professionals. The health professionals include doctors (71), professional nurses (74), pharmacists (15), (42) laboratory technicians and, in different wards, units and sections that are involved in the generation and handling of HCRW during performance of their daily duties

### 2.2. Population and Sampling

The population of the study were all the doctors, professional nurses, pharmacists and laboratory technicians aged 18 years and above. A representative sample from each category of healthcare professionals from different wards, units and sections was selected using stratified random sampling. A list of all health professionals was requested from the hospital’s Human Resource section and numbers were allocated to each category. Each number was written on a square piece of paper. The lottery or hat method was used where the numbered pieces of paper were placed in a container. Numbers were allocated to each category. Each number was written on a square piece of paper. The pieces of paper were mixed thoroughly followed by withdrawing numbered pieces of paper, until the desired sample size was reached. The “simple formula for proportions” developed by Yamane (1967) was used to calculate the sample size *n* = N/ (1 + N(e)^2^) which was n = 134 [22]. The “Published Tables” allows for a population ranging from 200–249 to have a sample size between 134–153 and in this case 154 was the sample size. However, the response rate was only 144 (94%).

#### Ethical Approval

The Ethics clearance certificate was granted by the University of Fort Hare Ethics Committee in April 2019 (Approval certificate Ref # 2019=04=004=MotlatlaMF). Permission to conduct the study at the tertiary hospital was obtained from the Northern Cape Department of Health and Chief Executive Officer of the tertiary hospital. All were informed about the study and its aims and objectives before the consent form was signed. The self-administered questionnaires with a unique number were handed over to them after signing the informed consent. Anonymity and confidentiality were ensured by asking participants not to write down their names or any personality identification on the questionnaires or envelopes of the completed questionnaires. The collected data were stored in a locked cupboard. The signed consent forms and completed questionnaires were stored separately in lockable cupboard. All scanned data are stored in a password-protected computer in accordance with the university of Fort Hare policy.

### 2.3. Data Collection

Self-administered questionnaires based on Johannessen, Dijkman, Bartone, Hanrahan, Boyer and Chandra (2000) [23] Health care waste management guidance notes were used to collect data. The questionnaire was divided into four sections: Demographic information, knowledge about the different types of HCRW, knowledge related to HCRW management systems and participants’ self-rating of their HCRW management knowledge levels.

### 2.4. Analysis of Data

The descriptive statistics and R Foundation for statistical computing was used for statistical analysis and for inferential statistics [24]. Pearson–Fisher Chi-square statistics exact procedure was used to test statistical difference between 95% confidence intervals and *p*-values (*p* < 0.05) [25]. The results are presented in tables. The Ethics clearance certificate was granted by the University of Fort Hare Ethics Committee and permission to conduct the study was granted by the Northern Cape Department of Health.

## 3. Results

### 3.1. Demographic Characteristics of Participants

The findings of this study indicates that a little more than 102 (70.83%) were 40 years old and below. The majority (130), which was 90.28%, of the participants were aged 19–50 years with only 14 (9.72%) aged above 50 years and also the same number had 10 and below years of experience. Among the 144 participants, a total of 102 (70.83%) were females and only 42 (29.17%) were males. The gender distribution of the participants was a true representation of gender demographics at the tertiary hospital. There were 52 professional nurses (36.11%), 39 laboratory technicians (27.08%), 38 doctors (26.39%) and 15 pharmacists (10.42%). The participants were from all the sections, wards and units of the hospital. The participants’ work experience ranged from one year to over thirty-one years with the majority 128 (88.89%) having one to twenty years work experience (Table 1). There was a high number (70.83%) of health professionals with one to ten years’ work experience and this could have had an impact on the management of HCRW.

### 3.2. Knowledge of Participants on the Types of HRCW Generated at the Tertiary Hospital

The majority (128) of the health professionals (88.89%) were aware that sharps are considered to be HCRW. However, more that 72% (104) were not aware that the general hospital refuse is not classified as HCRW but HCW. This could be an indication of inadequate knowledge on HCRW, which could be responsible for the mixing of HCRW with general hospital refuse. A total of 95 (65.97%) and 75 (52%) participants were aware that infectious non-anatomical waste and pharmaceuticals, respectively, are HRCWs. Interestingly, less than 30% of the health professionals were aware that cytotoxic (39 (27%) and radioactive (25 (17.6%)) are HCRW. All the lab technicians were not aware of the radioactive waste, but more than half (21 (53.86%)) of them were aware that cytotoxic waste was HCRW. This was interesting as 27 (69.23%) of the laboratory technicians were trained and one would have expected them to have more knowledge on types of HCRW. There were only 51 (35.42%) health professionals who indicated that they were trained on HCRW management and these were 27 laboratory technicians, 11 medical doctors, 10 professional nurses and three pharmacists (Table 2). The lack of training could be responsible for the challenges the tertiary hospital is facing regarding management of HCRW. This could be responsible for the high volumes of HCRW, as HCW segregation is not done properly and general waste becomes contaminated.

### 3.3. Knowledge of Participants on Available HCRW Legislative Documents at the Tertiary Hospital

Table 3 shows that the majority (84.62%) of the laboratory technicians were aware of the HCRW policy (33) and HCRW Standard Operating Procedure (SOP) (33), and about 71.79% were aware of the HCRW plan (28). These were followed by the 31 professional nurses who were aware of the policy (59.62%), eight doctors (21.05) and five pharmacists (33.33). A little more than half of the pharmacists (eight (53.3)) and half of the professional nurses (26 (50%)) and 12 (31.58%) doctors were aware of the HCRW SOPs in their hospital. Only 23 (44.23) of the professional nurses, seven doctors and six pharmacists were aware of the hospital HCRW plan. In essence more than half of the health professionals had no knowledge of the availability of the policy documents related to the management of the HCRW. Similarly, more than half of the health professionals were not aware of the existence of the waste management officer (WMO) in their hospital and where to report HCRW challenges in their wards or units. However, the majority of the laboratory technicians were aware that the hospital has a waste management officer and knew where to report any occupational injuries.

A similar pattern was observed on the knowledge of health professionals regarding the existence of an occupational health nurse (OHS) a little more than half 79 (55%) were aware. In other words, about 65 (45%) health professionals did not know where to report occupational injuries in the hospital should they need to report injuries on duty. However, the majority of the laboratory technicians knew where to report any occupational injuries or exposure to hazardous substances. Only 75 (52%) health professionals were aware of the presence of an infection prevention and control nurse (IPCN) at the hospital.

### 3.4. Knowledge about the Disposal Containers Used for HCRW

The health professionals were asked if they knew the type of waste container used for the different waste material produced at the hospital. Their knowledge of containers was used as an indication that the health professionals are able to segregate the HCW in the hospital appropriately. The majority (133), which is 92%, had knowledge that sharps waste was disposed in a yellow bin container, except eleven participants who had no knowledge. Among those who indicated the correct container, doctors (36 (94.73%)), professional nurses (49 (94.24%)), pharmacists (12 (80%)) and laboratory technicians (36 (92.3%)) knew that the sharps waste container should be mounted on the wall. The professional nurses (39 (75%)), laboratory technicians (22 (56.41%)), doctors (20 (52.63%)) and pharmacists (six (40%)) were aware that the red specibin was for anatomical waste disposal. With regard to the container used for the disposal of pharmaceutical waste, the pharmacists were the highest (12 (80%)) among all the health professionals, followed by the doctors at 23 (60.53%) and professional nurses at 28 (53.84%), and the laboratory technicians were the lowest (14 (35.89%)) in identifying the dark green container as the correct container for the disposal of pharmaceutical waste. The knowledge of health professionals on the disposal of the cytotoxic waste container showed varying answers and knowledge levels, with 20% of participants providing no response to the question. The red liner and box was identified as a cytotoxic waste container by only ten (26.3%) of the doctors, professional nurses 12 (23.07%), laboratory technicians five (12.82%) and only one pharmacist (6.67%) (Table 4).

### 3.5. Knowledge on Storage of HCRW Time Frames at the Hospital Storage Facility

Table 5 was related to the knowledge among professionals with regard to minimum time frames, as legislated, for HCRW at the facility before final disposal. Most of the health professionals were not aware of the period. Of the 144 participants, only 39 (27.08%) were aware that sharps waste should be stored for 90 days in the hospital storage facility. For infectious waste, only 42 (29.17%) indicated that the correct period for infectious waste storage is 72 h. Forty-four participants were aware that anatomical waste should only be stored for 24 h in the hospital storage and only 47 (32.64%) of the participants knew that pharmaceutical waste should be stored for 90 days.

### 3.6. Knowledge of Central Waste Storage

The findings in Table 6 sought to understand if the health professionals were aware of any person responsible for managing the central waste storage area, storage demarcations and if it was locked at all times. The results revealed the following: Doctors (20 (52,63%)), professional nurses (10 (65.38%)), pharmacists (eight (53.33%)) and laboratory technicians (28 (71.79%)) were aware that there was a person designated to manage the central storage area. The doctors (13 (34.21%)), professional nurses (25 (48.08%)), pharmacists (seven (46.67%)) and laboratory technicians (16 (41.03%)) indicated that the central waste storage was demarcated for different types of waste. Additionally, 21 (40.38%) of the professional nurses and 22 (56.41%) of the laboratory technicians indicated that the central waste storage was locked at all times; no response from doctors and pharmacists.

### 3.7. Participants’ Rating of the Tertiary Hospital’s Compliance with the HCRW Legislation, Guidelines and Standards

This section intended to present findings about how individual health professionals rated the tertiary hospital regarding its compliance with HCRW regulations and legislations, based on their observation of practices on HCRW management at the hospital. More than three quarters of the health professional participants 77 (77.08%) rated the tertiary hospital’s compliance with HCRW legislation and regulations very low. However, opinions of 18.42% of doctors, eight (15.38%) professional nurses, three (20%) pharmacists and 15 (38.46%) laboratory technicians were of the view that the HCRW management at the hospital was compliant with legislation and regulations all the time (Table 7).

### 3.8. Participants’ Rating of Their Own Knowledge Related to HCRW

More than half (74 (51.39)) of the participants rated themselves as having average knowledge about HCRW. These were 22 (57.89%) of the doctors, 25 (48.08%) of the professional nurses, nine (60%) of the pharmacists and 18 (46.15%) of the laboratory technicians. Additionally, 25.69% of the participants rated their own knowledge of HCRW as very low and low. These were as follows: Five (13.16%) and six (11.54%) of the doctors and professional nurses, respectively, rated their knowledge of HCRW as being very low. Whereas four (26.67%) and eight (21.05%) of the pharmacists and doctors, respectively, and eight (15.38%) of both professional nurses and laboratory technicians rated themselves as low. Participants who rated themselves as having very good knowledge of HCRW were three (5.77%) professional nurses and, mainly, five (12.82%) laboratory technicians (Table 8).

### 3.9. Test of Independence

In analyzing the relationship between cumulative knowledge and the different sets of variables involved in the study, the Pearson’s Chi-squared test and the Fisher’s exact test (with re-scaled simulated *p*-value based on 2000 replicates) were used. The results suggest that cumulative knowledge on temporal waste storage area and period of waste storage are dependent on professional category because their *p*-values for all the tests are less than the 5% significant level. On the confidence intervals for the odds ratios, a sample size of 144 were randomly selected from the population of the health professionals at the tertiary hospital on numerous occasions; the respective intervals will contain the estimated odds ratios in approximately 95% of the cases, assuming there are no biases or cofounding (Table 9).

### 3.10. Test for Independence: Knowledge Vs. Professional Category

Cumulative knowledge on period of waste storage is dependent on the gender of the professional because the chi-square and Fisher exact *p*-values are less than the 5% significant level, as reported by Table 10. This result is confirmed by the odds ratio, because the ratio is different from one. Since the odds ratio is greater than one, gender is negatively related to cumulative knowledge on period of waste storage.

### 3.11. Test for Independence: Knowledge Vs. Experience

In Table 11, the *p*-values for all knowledge types are greater than 0.05, with the exception of waste storage period. In addition, the corresponding odds ratios are approximately equal to one, except that of waste storage period, which is far more than one. These results suggest that, among all knowledge types, waste storage period is the only variable that is significantly related to experience and the nature of the relationship is positive.

## 4. Discussion

The demographic representation of the participants was important in the study in order for the author to extrapolate any association which may be observed and provide scientific conclusions thereof. The findings of this study indicates that 70.83% of the participants were 40 and less years of age, with a similar percentage having professional experience of ten years and below. This scenario may imply that the majority of the health professionals over the age of 40 years and above 10 years of experience have exited the health professional working group in the public sector or at the tertiary hospital. This may have an impact on the level of understanding regarding the HCRW management due to education and syllabus that has evolved over time. The third and fourth industrial revolution could have a negative or positive impact on the level of knowledge between different categories of age groups. Eradication or emergency of certain health conditions could have triggered new ways of applying knowledge. However, this is similar to the findings by Wandner, Heft, Lok, Hirsh, George, Horgas, Atchison, Torres and Robinson (2014) [26], where the average age of the healthcare providers was 44 years and the average years of professional experience was 15 years. Among the 144 participants, a total of 102 (70.83%) were females and only 42 (29.17%) were males. The gender distribution of the participants was a true representation of gender demographics at the tertiary hospital and is similar to that of the study by Wandner et al. (2014) [26], where females constituted 71% of the participants. The general observation between the two studies may require more research regarding knowledge of the new group of health professionals with regard to HCRW management, and if it is consistent with the latest emergency of health conditions and industrial revolution systems in place.

The findings revealed that 51 (35.52%) of the 144 participating health professionals in the study underwent HCRW management training, and the majority were laboratory technicians 51 (69.23). It is evident that the tertiary hospital at Northern Cape does not have a similar training program for different categories of health professionals and this may lead to poor segregation and non-compliant disposal of HCRW by other health professionals. The incorrect segregation often renders normal HCW to become HCRW, which requires costly treatment and creates a risk to all people coming in contact with it. This is similar to the findings at George Regional hospital, where they found that limited staff knowledge about HCRW has had an impact on waste segregation. They then embarked on staff training to ensure that staff gained the required knowledge to practice proper HCRW management. An improvement in HCRW management was observed [27]. Makhura, Matlala and Kekana (2016) [28] argue that adequate knowledge among health professionals is necessary to improve HCRW handling and disposal. They also argued that all health professionals receive training on the correct disposal of HCRW and HCW in general during their professional training. However, their recommendation was that hospital management must ensure that healthcare professionals receive continuing training in HCRW handling and disposal [28]. The recommended type of training is similar to that offered at the tertiary hospital in the Northern Cape, as it is also a continuing education program that is offered intermittently. Hence, a large number of the participants in this study had not had the opportunity to attend training. The World Health Organization (2020) [29] recommends that HCRW training should include hospital personnel, families and communities through a program that will build capacity among healthcare staff, families and communities on HCRW, in order for them to protect themselves and environment from exposure to and injuries from contaminated and polluted environment. However, there are arguments that indicate that knowledge alone cannot change behavior. There is a need; therefore, that HCRW training programs should also target behavior change to ensure sustainable behavior change among health professionals. This will lead to proper HCRW segregation, management and reduction of HCRW in healthcare facilities, particularly tertiary hospitals that offer many specialties [29].

Participants were asked to identify hospital waste that is considered to be HCRW. The majority (89%) of them correctly indicated that sharps are considered HCRW. About 66% and 76% participants had knowledge that infectious non-anatomical waste and pharmaceuticals, respectively, are HRCW. However, more that 72% of the participants were not aware that the general hospital refuse is not classified as HCRW. This could be an indication of limited knowledge on HCRW among health professionals. The challenge related to this limited knowledge is that the participants could be unknowingly mixing general hospital refuse with HCRW. The mixing of HCRW with general hospital waste is a serious challenge in many hospitals, including the Northern Cape tertiary hospital, because it causes high volumes of HCRW. According to Sartaj and Arabgol (2015) [30], this is a challenge that exists in many healthcare facilities due to lack of programs for waste minimization and poor hospital waste segregation leading to contamination of general waste; therefore, increasing the volume of HCRW. This is exacerbated by lack of HCRW appropriate treatment equipment and incinerators. Additionally, lack of trained health professionals as generators and handlers of HCRW and trained personnel for the HCRW storage facilities are responsible for poor hospital waste segregation. Proper waste segregation in health facilities requires knowledge about the different bins or containers used for general hospital waste and HCRW among health professionals and hospital storage facilities [30].

The majority of the participants at the tertiary hospital in the Northern Cape were aware of the bin for sharps and that the bin must be mounted on the wall. However, very few of these participants were aware that sharps should be disposed of when the container is three quarters full. These findings were higher than those of a study conducted at a tertiary hospital at Lucknow district, whereby only 66% of the health professionals reported to know the correct waste container for sharps waste disposal [31]. On average, about half of the Northern Cape tertiary hospital participants were aware of the bin for anatomical waste disposal and the bin for pharmaceutical waste, but very few managed to identify the bin for cytotoxic waste. This could be an indication that waste segregation at the tertiary hospital is done incorrectly, because health professionals who are the generators and handlers of HCRW have limited knowledge of the waste containers. The inadequate knowledge among professional could interfere with waste segregation at the generation level. It is argued that proper segregation practices depend on the knowledge of HCRW waste generators who are the healthcare providers [8,32]. A study conducted in rural clinics in the KwaZulu-Natal Province showed that, in some clinics, the healthcare professionals indicated correctly the different types of waste containers and where they are stored; however, upon inspection, they found that waste was mixed, which meant that the healthcare professionals were not practicing waste segregation as it should be done. In other words, these healthcare professionals had knowledge with no behavior change in their practice [28,33].

Although participants were aware of the containers for the different HCRW, less than 60% of them were not aware of the storage period of HCRW at hospital storage facility. It is important for the healthcare professionals and HCRW storage management to know the timeframes for storing different HCRW and to understand the impact that mismanaged HCRW storage can have within the hospital and community [34,35]. Proper separation of HCRW into designated categories is an important and crucial point in the waste management process because it determines the amount of waste and type of treatment process to which the HCRW should be subjected to [34]. According to Nwachukwu, Orji and Ugbogu, (2013) [1], health professionals and HCRW storage managers must be aware that HCRW contains potentially harmful micro-organisms which can infect hospital personnel, patients, and the general public. These micro-organisms can also spread drug-resistance into the environment. Some of the HCRW can cause injuries (e.g., radiation burns, sharps-inflicted injuries, and poisoning and pollution through pharmaceutical products and cytotoxic products). A study in Botswana found that the storage facilities and collection services in the healthcare facilities had serious challenges. They were not operating as effectively and efficiently as expected or in accordance with the policies [34].

Compliance with the legislation, policies and regulations at the Northern Cape tertiary hospital was rated as very low by the majority of the participants (77.08%). This raises concerns as the participants themselves also considered their own knowledge of HCRW as poor and very poor. In addition, more than half of the participants were not aware of the HCRW policy and the hospital HCRW plan. However, about 55% of them were aware of the SOPs.

The health professionals (≤55%) were aware of the presence of HCRW officials and the HCRW policies, SOPs and plan. The majority of these participants were laboratory technicians had received HCRW training. Similarly, more than 77% of health professionals rated the tertiary hospital as non-compliant with HCRW legislation and regulations. Interestingly, a similar percentage of the participants rated their own compliance to the policies, regulations and SOP during the performance of their duties as average, low and very low. Compliance of the tertiary hospitals with the policies, SOPs and regulations is dependent on the staff compliance with those policies, SOPs and regulations. It is; therefore, not surprising that the hospital is deemed non-compliant if the health professionals themselves, who are the main generators and handlers, are non-compliant. In contrast, a study in New Delhi showed that 72% of healthcare workers were aware of the biomedical waste management. The authors argue that one of the key strategies to improve HCRW management is increasing the number of HCRW0trained health professionals in the hospital [35]. However, in their study only 34% reported that they received HCRW management training at the hospital. This was lower than the findings on a study in Namibia, where HCW (43%) reported to have undergone HCRW training [36]. As already indicated above, training for knowledge only does not always ensure compliance in practice.

The demographic representation of the participants triggered the author to investigate the association of independent variables such as experience, gender and profession category which may have an impact on health professionals’ knowledge of HCRW management The results of the Northern Cape tertiary hospital show that the cumulative knowledge on the temporary waste storage area are dependent on professional category and gender. Gender and work experience were also found to positively influence cumulative knowledge on waste storage period. This result suggests that experienced professionals were more knowledgeable on the period for which HCRW are stored before disposal, whereas gender may have the upper hand on cumulative knowledge on waste storage period. The results of a similar study conducted among post-graduates, doctors, nurses, laboratory technicians and domestic workers in Lucknow district showed that there was association between waste segregation practices and age, gender, occupation status, work experience and training and it was statistically significant (*p* < 0.05) [31].

## 5. Conclusions

The study revealed that knowledge of health professionals on HCRW management is not evenly distributed among all categories of professionals. The majority of the participants had some knowledge of segregation of HCRW, particularly for disposal of sharps waste. However, limited knowledge was among the majority of participants about cytotoxic waste. This had an impact on segregation of waste and their compliance levels to legislation and regulations during the performance of their duties. This translated to non-compliance of the hospital. The lack of knowledge related to the policies, SOPs and plans is due to lack of training in the hospital. There is a need for behavior change training in order to ensure proper waste segregation and compliance to legislations and regulations governing HCRW, of which facilitate prevention of transmission of HIV, hepatitis B and C. In addition, the training in HCRW should include patients, families and communities to ensure that they are able to identify hazardous materials in their community and report as soon as possible to prevent exposing communities to injuries, environmental pollution and poisoning. The hospital management must ensure that HCRW-trained health professionals and HCRW management officials put into practice what they have learnt. Furthermore, the hospital should focus on training health professionals on HCRW storage periods in order to address additional issues, such as managing HCRW exposure levels on healthcare workers, patients and the public.

## Figures and Tables

**Table 1:** Demographic characteristics of the participants (*n* = 144).

Variable	Frequency (*n* = 144)	Percentage
Age		
Between 19–30 years old	69	47.92
Between 31–40 years old	33	22.92
Between 41–50 years old	28	19.44
51 and above years old	14	9.72
Gender		
Male	42	29.17
Females	102	70.83
Profession		
Medical doctors	38	26.39
Professional nurses	52	36.11
Pharmacists	15	10.42
Laboratory technicians	39	27.08
Radiologists/radiographers	0	0
Years of experience		
1–10 years	102	70.83
11–20 years	26	18.06
21–30years	9	6.25
31 and above	7	4.86
Received healthcare risk waste (HCRW) training on HCRW		
Medical doctors	11	28.00
Professional nurses	10	19.23
Pharmacists	3	20.00
Laboratory technicians	27	69.23

HCRW: health care risk waste.

**Table 2:** Knowledge on types of healthcare risk waste (HRCW) generated and training at the tertiary hospital (*n* = 144).

Waste Types	Doctors (*n* = 38)		Professional Nurses (*n* = 52)		Pharmacists (*n* = 15)		Laboratory Technicians (*n* = 39)		Total (*n* = 144)	
Types of Waste	Frequency (Freq.)	Percentage (%)	Freq.	%	Freq.	%	Freq.	%	Freq.	%
Sharps	38	100	50	96	11	73	29	74	128	88.89
General Refuse	32	84	40	77	11	73	21	54	104	72.22
Anatomical	25	66	26	50	5	33	19	48	75	52.08
Infectious Non- Anatomical	33	87	43	83	6	40	13	33	95	65.97
Pharmaceuticals	26	68	26	50	14	93	10	26	76	52.77
Radioactive waste	10	11	14	14	1	7	0	0	25	17.36
Cytotoxic	6	16	7	29	5	33	21	54	39	27.08
Training on healthcare risk waste (HCRW)	11	28.95	10	19.23	3	20	27	69.23	51	35.42

Freq.: Frequency; %: Percentage; HCRW: healthcare risk waste.

**Table 3:** Knowledge on healthcare risk waste (HCRW) legislative documents and officials at the tertiary hospital (*n* = 144).

Documents	Doctors (*n* = 38)		Professional Nurses (*n* = 52)		Pharmacists (*n* = 15)		Laboratory Technicians (*n* = 39)	
Healthcare Risk Waste (HCRW) Legislative Documents	Frequency (Freq.)	Percentage (%)	Freq.	%	Freq.	%	Freq.	%
HCRW Standard Operating Procedure (SOP)	12	31.58	26	50	8	53.33	33	84.62
HCRW Policy	8	21.05	31	59.62	5	33.33	33	84.62
HCRW Plan	7	18.42	23	44.23	6	40	28	71.79
HCRW officials at the tertiary hospital								
Officials	Freq.	%	Freq.	%	Freq.	%	Freq.	%
Waste Management Officer (WMO)	9	23.68	26	50	6	40	35	89.74
Occupational Health Nurse (OHN)	20	52.63	27	51.92	7	46.67	26	66.67
Infection Prevention Control Nurse (IPCN)	21	55.26	33	63.46	7	46.67	17	43.59
HCRW Representative	7	18.42	13	25	1	6.67	31	79.49
Total	57	149.99	99	190.38	21	140.01	109	279.49

Freq.: frequency; %: percentage; HCRW: healthcare risk waste; WMO: waste management officer; IPCN: infection prevention control nurse; OHN: occupational health nurse.

**Table 4:** Knowledge on HCRW container types at the tertiary hospital.

Waste Container Types	Doctors (*n* = 38)		Professional Nurses (*n* = 52)		Pharmacists (*n* = 15)		Laboratory Technicians (*n* = 39)		Total (*n* = 144)	
	Frequency (Freq.)	Percentage (%)	Freq.	%	Freq.	%	Freq.	%	Freq.	%
(Sharps) Yellow Bins	36	94.73	49	94.24	12	80	36	92.3	133	92.36
(Anatomical) Red Specibin	20	52.63	39	75	6	40	22	56.41	75	52.08
(Infectious) Red Liner and Box	20	52.63	29	55.77	3	20	9	23.08	95	65.97
(Pharmaceutical) Dark Green Bin	23	60.53	28	53.84	12	80	14	35.89	76	52.78
(Cytotoxic) Red Liner and Box	10	26.3	12	23.07	1	6.67	5	12.82	39	27.08

Freq.: Frequency; %: Percentage.

**Table 5:** Knowledge on HCRW storage period at the tertiary hospital.

Responses	Doctors (*n* = 38)		Professional Nurses (*n* = 52)		Pharmacists (*n* = 15)		Laboratory Technicians (*n* = 39)		Total (*n* = 144)	
Response (Yes)	Frequency (Freq.)	Percentage (%)	Freq.	%	Freq.	%	Freq.	%	Freq.	%
Sharps waste	9	23.68	17	32.69	2	13.33	11	28.21	39	27.08
Infectious waste	8	21.05	21	40.38	4	26.67	9	23.08	42	29.17
Anatomical waste	9	23.68	21	40.38	5	33.33	9	23.08	44	30.56
Pharmaceutical waste	9	23.7	24	46.2	10	66.7	4	10.3	47	32.64
Total	35	92.11	83	159.65	21	140.03	33	84.67	172	119.44

Freq.: Frequency; %: Percentage.

**Table 6:** Percentage distribution on knowledge of central waste storage area (*n* = 144).

Responses	Doctors (*n* = 38)		Professional Nurses (*n* = 52)		Pharmacists (*n* = 15)		Laboratory Technicians (*n* = 39)		Total (*n* = 144)	
Central waste storage area (Yes)	Frequency (Freq.)	Percentage (%)	Freq.	%	Freq.	%	Freq.	%	Freq.	%
Designated storage	25	65.79	38	73.08	10	66.67	37	66.67	110	76.39
Storeman	20	52.63	34	65.38	8	53.33	28	71.79	90	62.50
Demarcation	13	34.21	25	48.08	7	46.67	16	41.03	61	42.36
Lock and key	5	13.16	21	40.38	5	33.33	22	56.41	53	36.81
Total	63	165.79	118	226.92	30	200	103	235.9	314	218.06

**Table 7:** Percentage distribution of health professionals’ own rating of knowledge on HCRW legislation (*n* = 144).

Response	Doctors (*n* = 38)		Professional Nurses (*n* = 52)		Pharmacists (*n* = 15)		Laboratory Technicians (*n* = 39)		Total (*n* = 144)	
Health care risk waste (HCRW) legislation	Frequency (Freq.).	Percentage (%)	Freq.	%	Freq.	%	Freq.	%	Freq.	%
Always	7	18.42	8	15.38	3	20	15	38.46	33.00	22.92
Sometimes	17	44.74	29	55.77	8	53.33	15	38.46	69.00	47.92
Never	2	5.26	5	9.62	1	6.67	0	0	8.00	5.56
Do not know	12	31.58	9	17.31	3	20	9	23.08	33.00	22.92
Total	38	100	51	98.08	15	100	39	100	143.00	99.31

Freq.: frequency; %: percentage; HCRW: healthcare risk waste.

**Table 8:** Percentage distribution of health professionals’ own rating of knowledge on HCRW management (*n* =144).

Response	Doctors (*n* = 38)		Professional Nurses (*n* = 52)		Pharmacists (*n* = 15)		Laboratory Technicians (*n* = 39)		Total (*n* = 144)	
Own rating	Frequency (Freq.)	Percentage (%)	Freq.	%	Freq.	%	Freq.	%	Freq.	%
Very low	5	13.16	6	11.54	0	0	0	0	11	7.64
Low	8	21.05	8	15.38	4	26.67	6	15.38	26	18.06
Average	22	57.89	25	48.08	9	60	18	46.15	74	51.39
Good	3	7.89	10	19.23	2	13.33	10	25.64	25	17.36
Very good	0	0	3	5.77	0	0	5	12.82	8	5.56
Total	38	100	52	100	15	100	39	100	144	100.00

Freq.: Frequency; %: Percentage.

**Table 9:** Test for independence: Knowledge vs. professional category.

Knowledge vs. Professional Category	Chi-square		Fisher Exact
	Test Statistic	*p*-value	*p*-value
Container types for healthcare risk waste (HCRW)	3.9579	0.2789	0.2616
Temporary storage area	29.606	0.0005*	1.59 × 10^−6^*
Period of HCRW storage	21.028	0.0005*	9.42 × 10^−5^*

HCRW: healthcare risk waste.

**Table 10:** Test for independence: Knowledge vs. gender.

Knowledge vs. Gender	Chi-Square		Fisher Exact		
	Test Statistic	*p*-Value	*p*-Value	Odds Ratio	95% Confidence Interval
Container types for healthcare risk waste (HCRW)	0.0659	0.8596	0.8532	1.0485	(0.7173, 1.5306)
Temporary storage area	0.0526	0.8376	0.8457	0.9149	(0.4010, 2.1453)
Period of HCRW storage	6.7177	0.0135*	0.0119*	1.6519	(1.1051, 2.4617)

HCRW: healthcare risk waste.

**Table 11:** Test for independence: Knowledge vs. experience.

Knowledge vs. Experience	Chi-Square		Fisher Exact		
	Test Statistic	*p*-Value	*p*-Value	Odds Ratio	95% Confidence Interval
Container types for healthcare risk waste (HCRW)	1.1753	0.3248	0.3106	1.2452	(0.8206, 1.8915)
Temporary storage area	0.2571	0.6842	0.6761	0.8129	(0.3403, 2.0130)
Period of waste storage	13.448	0.0010*	0.0004*	2.0042	(1.3524, 2.9684)

HCRW: healthcare risk waste.

## References

[1] Chuks N., Anayo F., Ugbogu O.C. (2013). Health Care Waste Management—Public Health Benefits, and the Need for Effective Environmental Regulatory Surveillance in Federal Republic of Nigeria. Curr. Top. Public Health.

[2] WHO (2014). Safe Management of Waste from Health-Care Activities, 2nd ed. http://www.healthcarewaste.org.

[3] Olaniyi F.C., Ogola J., Tshitangano T.G. (2019). Efficiency of Health Care Risk Waste Management in Rural Healthcare Facilities of South Africa: An Assessment of Selected Facilities in Vhembe District, Limpopo Province. Int. J. Environ. Res. Public Health.

[4] Nyathi S., Olowoyo J.O., Oludare A. (2018). Perception of Scavengers and Occupational Health Hazards Associated with Scavenging from a Waste Dumpsite in Pretoria, South Africa. J. Environ. Public Health.

[5] Semenya K., Moja S.J. Assessment of Health Care Waste Management in Some Medical Facilities in Pretoria, South Africa. Proceedings of the 23rd WasteCon Conference.

[6] Abah S.A., Ohimain E.I. (2011). Healthcare waste management in Nigeria: A case study. J. Public Health Epidemiol..

[7] Johnson K.M., González M.L., Dueñas L., Gamero M., Relyea G., E Luque L., Caniza M.A. (2013). Improving waste segregation while reducing costs in a tertiary-care hospital in a lower–middle-income country in Central America. Waste Manag. Res..

[8] Olaniyi F.C., Ogola J.S., Tshitangano T.G. (2018). A Review of Medical Waste Management in South Africa. Open Environ. Sci..

[9] National Department of Environmental Affairs and Tourism (NDEAT) (2011). National Waste Management Strategy.

[10] National Environment Management (2008). Waste Act of South Africa (NEMWA). Waste Act. No. 59 of 2008 (No. 32000).

[11] National Health Act (Act No 61 of 2003) of South Africa (NHA) (2003). Regulations Relating to Categories of Hospitals (No. 35101) R185.

[12] National Department of Health (NDOH) (2003). Discussion Document: Strategic Framework for Modernisation of Tertiary Services. Civitas Room 2420.

[13] Republic of South Africa (1996). Constitution of the Republic of South Africa.

[14] Northern Cape Department of Health (NCDOH) (2016). Follow-Up Inspection of Kimberley Hospital Medical Waste at Sol Plaatje Landfill Site and Kimberley Hospital, Northern Cape Province.

[15] South African Local Government Association (SALGA) (2011). Northern Cape Municipalities. http://www.salga.org.za/pages/AboutSalga/./SALGA.

[16] Kimberley Hospital (2017). Community Servers and Interns.

[17] Komilis D.P., Fouki A., Papadopoulos D. (2012). Hazardous medical waste generation rates of different categories of health-care facilities. Waste Manag..

[18] Gai R., Kuroiwa C., Xu L., Wang X., Zhang Y., Li H., Zhou C., He J., Tang W. (2009). Hospital medical waste management in Shandong Province, China. Waste Manag. Res..

[19] Asante P., Amoako E.E., Denteh S.N. (2018). Assessment of Hospital Solid Waste Management in Tamale Metropolis: A Case Study of Tamale West and Central Hospitals. Int. J. Waste Resour..

[20] Mahasa P.S., Ruhiiga T.M. (2014). Medical Waste Management Practices in North Eastern Free State, South Africa. J. Hum. Ecol..

[21] United Nations Development Programme (UNDP) (2015). Sustainable Development Goals. Responsible Cities and Communities. https://www.undp.org/content/undp/en/home/sustainable-development-goals.html.

[22] Singh A.S., Masuku M.B. (2014). Sampling Techniques and Determination of sample size in applied statistical research: An overview. Int. J. Econ. Commer. Manag..

[23] Johannessen L.M., Dijkman M., Bartone C., Hanrahan D., Boyer M.G., Chandra C. (2000). Health Care Waste Management Guidance Notes. Health Nutrition and Population.

[24] Team R.C. (2019). R: A Language and Environment for Statistical Computing.

[25] Balboaca S.D., Jantschi L., Setras F.A., Setras R.E., Pamfil D.C. (2011). Peason’s–Fisher Chi-Square Statistics Revisited. Information.

[26] Wandner L.D., Heft M.W., Lok B.C., Hirsh A.T., George S.Z., Horgas A.L., Atchison J.W., Torres C.A., Robinson M.E. (2014). The impact of patients’ gender, race, and age on health care professionals’ pain management decisions: An online survey using virtual human technology. Int. J. Nurs. Stud..

[27] Global Green & Health Hospitals (2019). Reducing the Environmental Impact of Providing Healthcare.

[28] Makhura R.R., Matlala S.F., Kekana M.P. (2016). Medical waste disposal at a hospital in Mpumalanga Province, South Africa: Implications for training of healthcare professionals. SAMJ.

[29] World Health Organization (2020). Training Package for Health Care Providers.

[30] Sartaj M., Arabgol R. (2014). Assessment of healthcare waste management practices and associated problems in Isfahan Province (Iran). J. Mater. Cycles Waste Manag..

[31] Imchen T., Kumari R., Singh J.V., Srivastava K., Singh A. (2017). Study of biomedical waste management among healthcare personnel at a Tertiary hospital in Lucknow district. Int. J. Commun. Med. Public Health.

[32] Awodele D., Adewoye A.A., Oparah A.Z. (2016). Assessment of medical waste management in seven hospitals in Lagos, Nigeria. BMC.

[33] Gabela S.D., Knight S.E. (2010). Healthcare waste management in clinics in a rural health district in KwaZulu-Natal. South Afr. J. Epidemiol. Infect..

[34] Mmereki D., Baldwin A., Li B., Liu M. (2017). Healthcare waste management in Botswana: Storage, collection, treatment and disposal system. J. Mater. Cycles Waste Manag..

[35] Sharma P., Jais M., Gupta P., Ansari S., Lall H., Debbarma M., Kaur R. (2016). Awareness regarding biological waste management among healthcare workers in a tertiary care hospital in New Delhi, India. Int. J. Dev. Res..

[36] Haifete N.A. (2016). Knowledge, Attitude and Practice of Healthcare Workers on Waste Segregation at Two Public Training Hospitals, in Khomas Region, Namibia.

